# Evaluation of a Novel Automated Cerebral Ventricular Drainage System for Intracranial Pressure Monitoring and Cerebrospinal Fluid Drainage in Neurocritical Care Patients: A Prospective, Randomized Clinical Study

**DOI:** 10.1007/s12028-026-02477-4

**Published:** 2026-03-23

**Authors:** Josefine Maria Öhman, Niklas Marklund, David Cederberg

**Affiliations:** 1 Department of Clinical Sciences Lund, Lund University, Sölvegatan 19, Lund, 221 84 Skåne, Sweden; 2 Department of Neurosurgery, Skåne University Hospital, Entrégatan 7, Lund, 221 85 Skåne, Sweden

**Keywords:** Critical care, Intracranial pressure, Cerebrospinal fluid, Drainage

## Abstract

**Background:**

Neurointensive care is moving toward more automated patient management. This study aimed to evaluate a novel external ventricular drainage (EVD) system, the VGuard® system, as a tool for intracranial pressure (ICP) monitoring and automating ventricular drainage of cerebrospinal fluid (CSF), focusing on its safety and potential to reduce complications associated with manual EVD management.

**Methods:**

This prospective, randomized clinical study was conducted in the Department of Neurosurgery at Skåne University Hospital, Lund, Sweden. Patients (aged > 18 years) with different acute brain pathologies requiring an EVD were enrolled and randomized (1:1) to receive the VGuard® system or a manual EVD. Both systems employed a ventricular probe for simultaneous intraventricular and intraparenchymal ICP monitoring. Measurement accuracy was determined by comparing the median of absolute difference in ICP, using the Mann–Whitney *U* test. To further investigate the accuracy of ICP measuring, a post hoc Spearman correlation test was applied. In addition, as part of a post hoc analysis, we evaluated patient outcomes.

**Results:**

A total of 30 patients were included in the study. The VGuard® system demonstrated significantly better accuracy in ICP measurements compared with the manual EVD; median absolute difference: 1.07 versus 2.88, *p* < 0.001; median Spearman correlation coefficients: 0.81 versus 0.48, *p* < 0.05. There were no statistically significant differences in patient outcomes [mortality, Glasgow Outcome Scale-Extended (GOSE), modified Rankin Scale (mRS), median time with the EVD, and length of intensive care unit (ICU) stay], or adverse events (AE). No serious adverse events were noted.

**Conclusions:**

This is the first study to evaluate the VGuard® system, showing results of increased accuracy in measured ICP compared with the manual EVD. The VGuard® system offers an automated EVD solution, representing a step toward increased neurointensive care automation.

**Supplementary Information:**

The online version contains supplementary material available at 10.1007/s12028-026-02477-4.

## Introduction

Patients treated in a neurocritical care unit (NICU) often have various types of brain pathologies, for many of which placement of an external ventricular drain (EVD) into the ventricles of the brain is part of the treatment algorithm [1–5]. The EVD can be used to drain cerebrospinal fluid (CSF) and to measure the intracranial pressure (ICP) [2–8]. Draining CSF is one way to manage ICP. For ICP measurement, a pressure transducer, situated at the stand of the EVD, is used. The pressure transducer measures ICP via the fluid-filled tubing, connecting the ventricular catheter to a drip canister situated on the side of the patient [8–11]. When the ICP is higher than the level of the drip canister, CSF will drain. For the ICP measurements to be reliable a reference point must be defined. The most used reference point is equivalent to the midpoint of the brain, i.e., the foramen of Monro [8–12]. The ICP measurements will only be reliable if the pressure transducer is constantly kept at level with this position, referred to as the zero-level. It is important to register the correct ICP, to avoid potentially dangerous over- or underdrainage of CSF [13–16]. A laser pointer is often used to manually zero-level the pressure transducer when a patient’s head position is changed [8, 9, 14–16]. The nursing staff usually perform the zero-leveling, which can be a time-consuming task, particularly when the patient is mobile or confused.

To avoid under- or overdrainage of CSF due to incorrect zero-leveling, a novel automated ventricular drainage system was developed—the VGuard® system. The VGuard® system was designed to ensure that the pressure transducer follows the patient’s head, thus giving an accurate ICP measurement and ensuring that the correct amount of CSF can be drained. The system utilizes a fluid-filled tubing with a pressure sensor in each end. The external sensor is fixed to the patient’s head and connected to a motorized column situated at the stand of the EVD, automatically keeping the zero-level aligned.

Neurointensive care is moving toward more automated patient management [17–22]. However, automation of routine bedside care in neurointensive units remains limited. Placement of an EVD is among the most common neurosurgical procedures; however, to our knowledge, only one automated EVD system exists, the LiquoGuard® system (Möller Medical GmbH, Fulda, Germany), which monitors CSF pressure and flow, while VGuard® primarily focuses on ICP monitoring [23–26]. However, to our knowledge, the LiquoGuard® has not been evaluated in a randomized trial. Therefore, further development of the EVD, with a focus on automating the system, is essential. The aim of this randomized study was to evaluate a novel automated cerebral ventricular drainage system (the VGuard® system) focusing on its safety as a clinical device and how it compares to the current manual EVD. In addition, as part of a post hoc analysis, we evaluated patient outcomes.

## Methods

This study is a prospective, randomized, single-center, clinical study conducted at the Neurosurgery department at Skåane University Hospital, Lund, Sweden (ethics approval: Dnr 2021-04061) (Swedish Medical Council: CIV-SE-21-06-036906). The allocation ratio was 1:1. The study was monitored by Clinical Studies Sweden and preregistered at www.clinicaltrials.gov (NCT05177692) (2021/12/14). The study was performed in accordance with the ethical principles in the Declaration of Helsinki of 1975. Patient demographics were collected from the participants’ medical charts. ICP-data, adverse events (AE), and serious adverse events (SAE) were collected between December 2021 and March 2024. An AE was defined as an unexpected medical condition that was neither caused by the patient’s initial cause of admission to the NICU, nor as a consequence of the given intensive care unit (ICU) care. An SAE was defined as a participant’s death. As a post hoc analysis, patient outcomes were measured by mortality, Glasgow Outcome Scale-Extended (GOSE), modified Rankin Scale (mRS), median time with the EVD, and length of ICU stay in Region Skåne. The length of stay in the ICU and the median time of EVD were collected from the medical records of the participants. Mortality, GOSE and mRS were collected via phone and medical records after 6–18 months upon completion of study participation. Large language models (ChatGTP) were used for editorial work, such as word translation, grammar correction, and paraphrasing from the authors’ native language into English. All content was critically reviewed and verified by the authors.

### Study Population and Enrollment Procedure

The study population consisted of adult patients treated with a ventricular drain combined with an intraparenchymal ICP sensor, a Spiegelberg Tunnelling Silverline® ventricular probe (Spiegelberg GmbH & Co. KG, Hamburg, Germany). The inclusion criteria were: adults of either sex (> 18 years old) and treated at the NICU at Skåne University Hospital in Lund for a pathology requiring a ventricular drain with a combined intraparenchymal ICP sensor. It is important to note that while the clinical trial protocol originally specified an age range of 18–80 years, this was modified in the present study by removing the upper age limit. The exclusion criterion was that no consent could be obtained. The procedure of enrollment was led by the study coordinator or a person authorized by the study coordinator at the time the patient was admitted to the NICU. If the patient was deemed capable of deciding whether to participate in the study, consent was obtained directly from the patient. If not, consent was sought from the patient’s next of kin. After consent was obtained, participants were randomized to either the VGuard® system (the intervention group) or a manual EVD (the control group) by an envelope-based randomization process developed by Clinical Studies Sweden, an unbiased national collaboration between Sweden’s six healthcare regions, supported by the Swedish Research Council. Blinding was not feasible owing to the setup of the VGuard® system, which could not be masked. Patient recruitment was planned to continue until at least 14 participants in each group had been included. The estimated mean difference was 5 ± 2 mm Hg in the control group and 2 mm Hg in the intervention group. A sample size of 14 patients per group provided 80% power to detect a statistically significant difference at *p* < 0.05, with an allocation ratio of 1:1.

### Procedures of ICP Monitoring and CSF Drainage

The Spiegelberg ventricular probe enabled ICP measuring from both the brain parenchyma (referred to as IC1) and the ventricular drain (referred to as IC2). The ICP measured from the brain parenchyma (IC1) was considered as reference since this measurement was not dependent upon zero-leveling. In this study, the majority of drains remained open during ICP measurement, as it quickly became evident that clamping the drain was unfeasible in a real-world clinical setting. Thus, when draining CSF at the same time as the ventricular ICP was registered, the values of ICP may have been incorrect. Therefore, in this study, the intraparenchymally measured ICP was used as a reference, knowingly diverting from the common gold standard of ICP monitoring via the ventricles. The two ICP values enabled investigation of differences between IC1 and IC2. Thereafter, comparisons were made between the group of participants randomized to either the VGuard® system (intervention group) or a manual EVD (control group).

The EVDs were surgically placed in accordance with standardized guidelines, targeting the anterior horn of the right lateral ventricle when possible. [1, 8–11]. The primary head computed tomography (CT) was used to plan trajectory and to measure the depth from tabula externa to the intended ventricle target. All ICP values were recorded using Moberg Component Neuromonitoring System (CNS) (Moberg Research Inc, PA, USA). Data were collected in hertz per milliseconds and then converted to means per minute via the software Envision (Sep 2022 version) and exported to Excel (version 16.88). The decision whether or not to drain CSF was made in all participants individually according to standard treatment algorithms based on the underlying pathology.

### Description of the VGuard® System

**Fig. 1:**
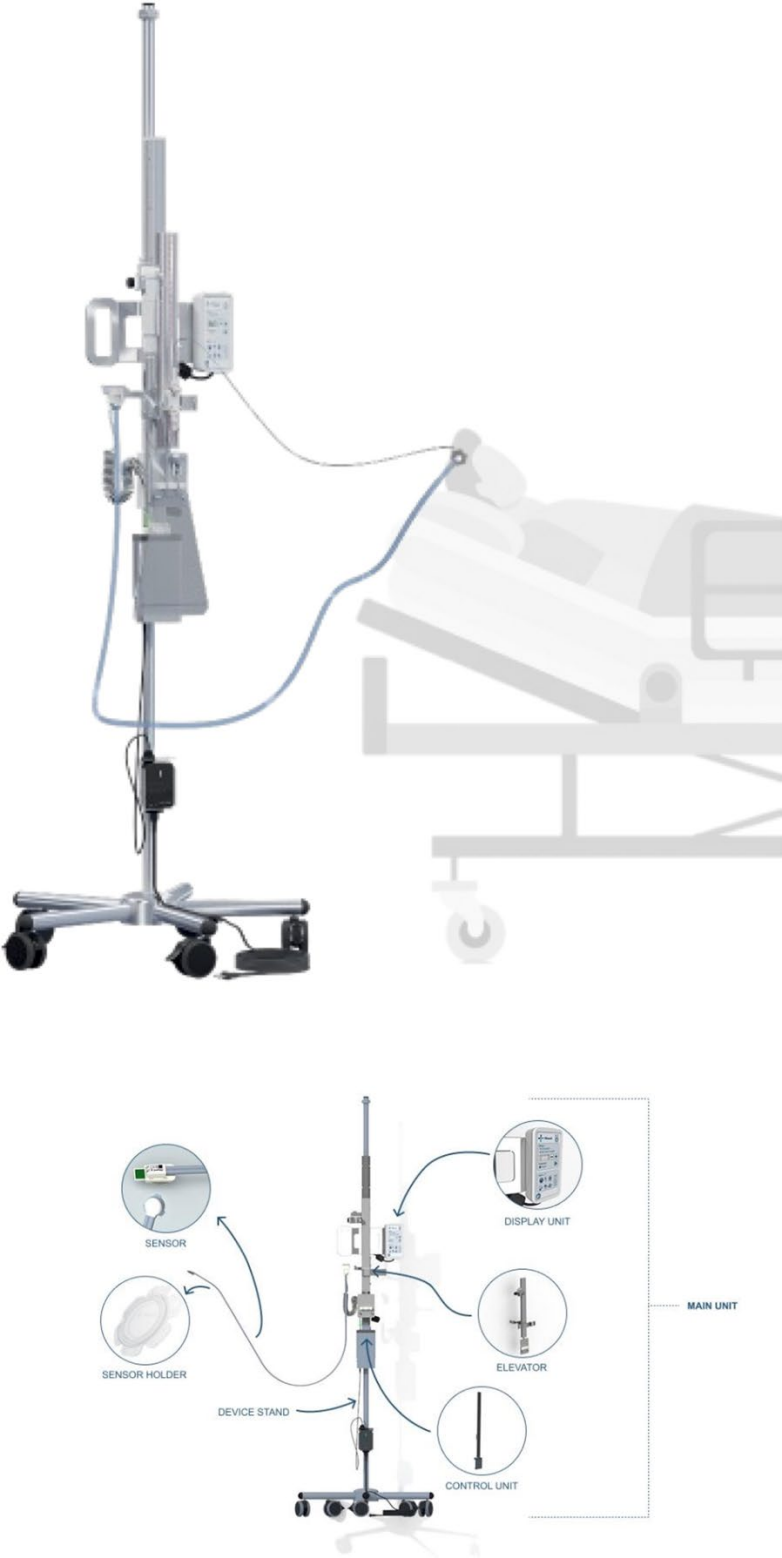
Presentation of the VGuard® system and its different components. The external ventricular drainage (EVD) with its different components is situated at the motorized column of the VGuard® system. The motorized column moves in a vertical direction, constantly keeping the pressure transducer in line with the midpoint of the brain, i.e., the foramen of Monro. The vertical movement of the column is achieved through a fluid-filled tubing with a pressure sensor in each end. The sensor is placed somewhere on the coronal suture and then connected to the motorized column, enabling the VGuard® system to automatically zero-level the EVD

The VGuard® system allows for simultaneous ICP measuring and CSF drainage. What differs the VGuard® system from the manual EVD is its ability to automatically zero-level the EVD, enabling correct ICP values to be measured. This is achieved through a fluid-filled tubing with a pressure sensor in each end. The sensor fixed to the participant’s head contains an accelerometer that takes into account the distance between the sensor and the tip of the ventricular drain. By placing the sensor somewhere on the coronal suture, the distance between the skin and the ventricle could easily be measured on the CT scan of the participant. The distance is put into the VGuard® system’s control panel. The range of motion of the VGuard® system is 70 cm. The system also contains an accelerometer. The accelerometer enabled the VGuard® system to adjust for small rotations and adjustments of the head. Should the accelerometer fail, the ICP values would consistently deviate by more than 5–10 mm Hg, at which point the VGuard® system would be disconnected (Supplementary Material). Should the accelerometer malfunction, it would not be replaced, only disconnected. The EVD stand is attached to a motorized column that automatically keeps the zero-point at level with the tip of the ventricular catheter at all times (Fig. 1). The system provides an alarm signal if it detects an error, such as power failure. This informs the nursing staff of a potential error, facilitating possible measures to be taken.

### Statistical Analysis

All data were analyzed in R software (version 2024.04.2 + 764). Quantitative numerical variables were tested for normal distribution by plotting histograms and QQ plots for visual determination, in combination with performed statistical tests for normality, such as the Kolmogorov–Smirnov test (*n* > 50), the Shapiro–Wilk test (*n* < 50), and the Anderson–Darling test to include additional evidence of normal distribution. After determining the data distribution, relevant statistical tests were applied. Significance level was set at *p* < 0.05. Patient demographics (baseline characteristics) were presented with descriptive statistics along with suitable effect sizes, confidence intervals, and *p*-values. For numerical variables, a Wilcoxon rank-sum test was applied. For categorical variables, a Fisher’s exact test was applied.

To investigate the accuracy of ICP measuring, an absolute difference between IC1 and IC2 was calculated every minute the Moberg component had registered an IC1 and IC2. An additional dataset was generated in which clinically irrelevant ICP values were excluded, such as measurements obtained while the drain was open, during recalibration of the intraparenchymal sensor or arising from electronic noise introduced by the Moberg monitor. A threshold-based filtering approach was used. The threshold values were determined with the help of previous literature as well as clinical experience [27–31]. A normal ICP in adults is typically around 5–15 mm Hg [28]. However, a normal ICP is hard to define, and several suggestions have been presented, such as < 10 mm Hg by Miller et al. (1977); < 15 mm Hg by Marshall et al. (1979); between 8 and 9 mm Hg by Hawryluk et al. (2020); and < 10–15 mm Hg by Tripathy et al. (2019) [27, 32–34]. Traditionally, an ICP > 20 mm Hg was regarded as intracranial hypertension, requiring treatment [35]. However, the most recent addition to the Brain Trauma Foundation (BTF) guidelines pushed this upper limit to 22 mm Hg [28]. In clinical practice, higher subthresholds are used. Generally, ICP values above 40–50 mm Hg are life threatening. In an article by Young et al. (2003), they investigated whether an upper ICP limit exists [30]. They reported several trauma patients who survived extremely high ICP values (40–50+ mm Hg) and reported values up to > 60–75 mm Hg. In a case report by Cederberg et al. (2020), a patient presented with an ICP > 90 mm Hg while still being fully awake [36]. This shows how uncertain the normal upper ICP limit is. However, in some physiological conditions ICP can approach 0 mm Hg or lower during, e.g., excessive CSF drainage or upright positions [29]. All calculations and statistical tests were applied to both the filtered and the non-filtered dataset. To explore differences in the calculated absolute difference in ICP between the two groups, a Mann–Whitney *U* test (MWU-test) was applied and reported alongside effect sizes as the rank-biserial correlation (*r*_*rb*_) with confidence intervals.

To further investigate the accuracy of ICP measuring, a post hoc Spearman correlation test was executed between IC1 and IC2 for each participant in the respective groups. The coefficients were then converted to *Z*-values by performing a Fisher *Z*-transformation. A median of *Z*-values was then calculated for each group and then reversed to a correlation coefficient by applying the inverse Fisher *Z*-transformation. All calculations and transformations were executed in R. A MWU-test was applied to the groups of *Z*-values, enabling investigation of differences in medians between the intervention group and the control group. The MWU-test is reported alongside effect sizes as the rank-biserial correlation with confidence intervals.

To explore differences in AE and SAE between the two respective groups, a Fisher’s exact test was applied and reported alongside effect sizes as odds ratio and confidence intervals. Variables used to describe patient outcomes were analyzed regarding differences and associations between the intervention group and the control group. For numerical variables, an MWU-test was applied. A shift analysis was applied to analyze the modified Rankin Scale (mRS). A Fisher’s exact test was applied when investigating differences in mortality among the two groups. Additionally, effect sizes were reported as the rank-biserial correlation for numeric variables and as the odds ratio for categorical variables with confidence intervals.

## Results

### Patient Demographics and General Findings

**Fig. 2:**
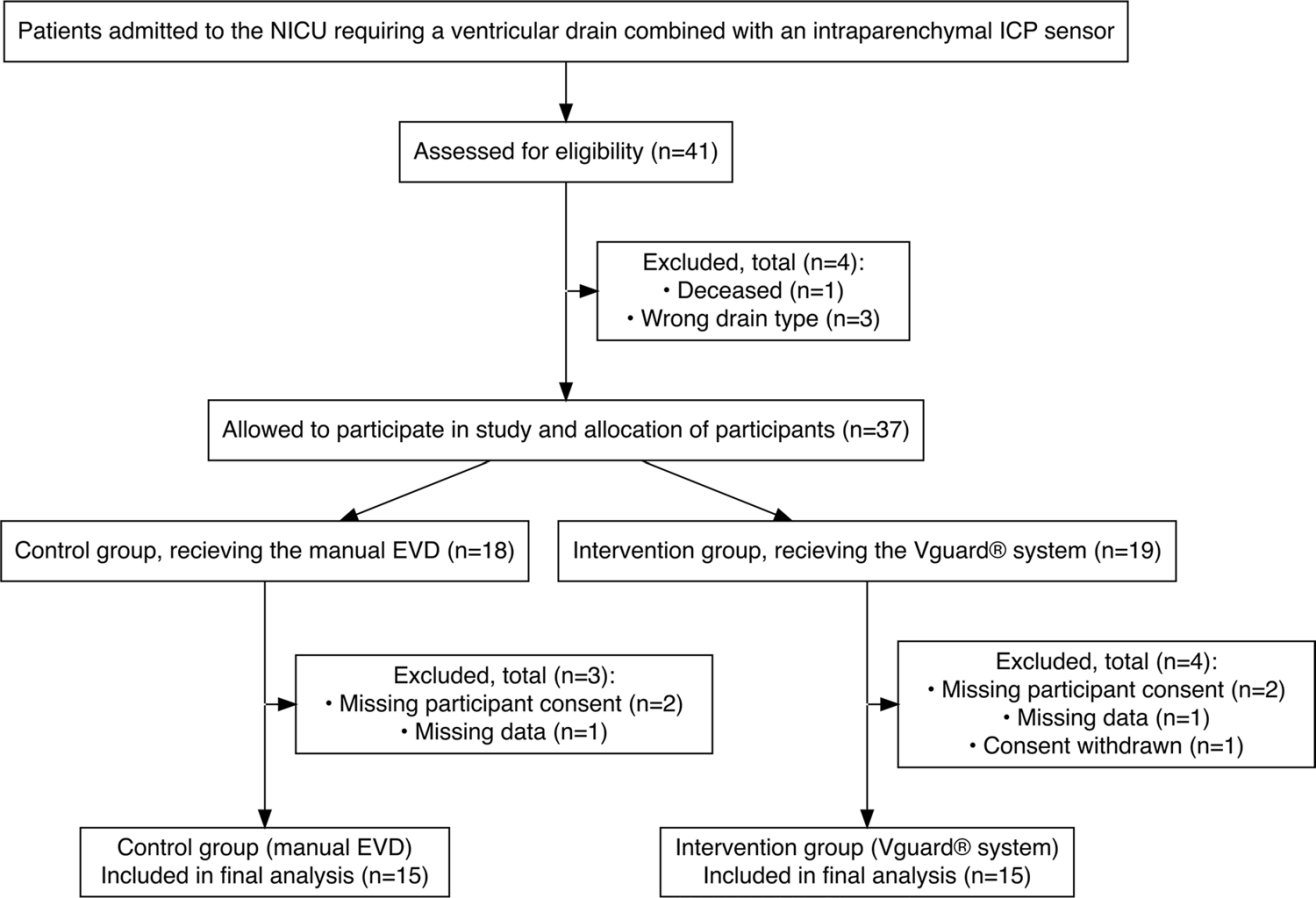
Flowchart diagram of the enrollment and allocation process. Patients admitted to the neurointensive care unit requiring a ventricular drain combined with an intraparenchymal ICP sensor were included. After assessing 41 patients for eligibility, 4 were excluded. In accordance with the inclusion and exclusion criteria, 37 patients were allowed to participate. Subsequently, the participants were randomized by envelope to either the intervention group or the control group. Finally, 15 participants were included in each group. The first participant was enrolled in December 2021 and the last participant was recruited in March 2024

A total of 30 patients were included in the final analysis, with 15 participants in each group. Power calculations indicated that 14 participants per group (1:1) would yield 80% power at *p* < 0.05. Two extra participants were initially enrolled as a buffer, but enrollment was ultimately capped at 15 participants per group. For more details regarding the enrollment and allocation process, please see Fig. 2. Concerning baseline characteristics, the two groups were comparable. The median age was 66 years in the intervention group and 63 years in the control group. The median initial motor component of Glasgow Coma Scale (mGCS) was 4 in both groups. The dominant admission diagnosis was subarachnoid hemorrhage (SAH) (80%) in both groups. Please see Table 1 for more details regarding patient demographics.

**Table 1:** Demographic data of study participants

Demographic variable		Control group	Intervention group	Effect size [95% CI] ; *p*-Value
Number of participants		15	15	
Median age, years (range)		63 (24–75)	66 (20–82)	0.20 [−0.22, 0.55] ; 0.37^1^
Sex (male (%): female (%))		9 (60%): 6 (40%)	6 (40%): 9 (60%)	−0.20 [−0.49, 0.14] ; 0.47^2^
Median initial mGCS^3^ (IQR)		4 (3.5–5)	4 (3.5–5)	−0.06 [−0.44, 0.35] ; 0.80^1^
Admission diagnosis (*n* (%))	Subarachnoid hemorrhage	12 (80%)	12 (80%)	0 [−0.28, 0.28] ; 1.00^4^
	Traumatic brain injury	1 (7%)	2 (13%)	0.07 [−0.18, 0.32] ; 1.00^4^
	Bacterial meningitis	2 (13%)	0 (0%)	−0.13 [−0.38, 0.09] ; 0.48^4^
	Posterior fossa hemorrhage	0 (0%)	1 (7%)	0.07 [−0.14, 0.30] ; 1.00^4^
Previous medical history (*n* (%))	Cardiovascular disease	12 (80%)	12 (80%)	0 [−0.28, 0.28] ; 1.00^4^
	Cerebrovascular accident	1 (7%)	2 (13%)	0.07 [−0.18, 0.32] ; 1.00^4^
	Diabetes	3 (20%)	2 (13%)	−0.07 [−0.34, 0.21] ; 1.00^4^
	Hematological disease	0 (0%)	1 (7%)	0.07 [−0.14, 0.30] ; 1.00^4^
	Non-CNS tumor	2 (13%)	1 (7%)	−0.07 [−0.32, 0.18] ; 1.00^4^

^1^Effect size as rank-biserial correlation with 95% CI. *p*-Value by the Wilcoxon rank-sum test

^2^Effect size as risk difference (RD) (RD = Pintervention − Pcontrol) with 95% CI, event = male. *p*-Value by Fisher’s exact test

^3^Motor component of Glasgow Coma Scale

^4^Effect size as risk difference (RD) (RD = Pintervention − Pcontrol) with 95% CI, event by categories. *p*-Value by Fisher’s exact test

### ICP Measurements and Results

In the intervention group, a total of 148 days (∼3552 h) of ICP monitoring was recorded versus 113 days (∼2712 h) in the control group. The median number of days for participation in the study was approximately 10 days in the intervention group and 6 days in the control group. Some recorded data points had a registered IC1 value but not a registered IC2 value and vice versa. If that was the case, those data points were excluded, resulting in a total of 1914 data points being removed from the intervention group with an original 185,713 existing data points per minute. In the control group, a total of 1872 data points were removed from the original data with 135,638 data points per minute. Filtering the data with the help of threshold values set to −15 ≥ IC1 ≤ 60, −15 ≥ IC2 ≤ 60 and absolute difference ≤ 20 resulted in 2634 data points being excluded from the data in the intervention group and 2420 data points in the control group. Additionally, the distance from the tip of the catheter to the outer tabule (tabula externa) was measured on the post-implantation CT. In the control group, the median distance was 6.67 cm [interquartile range (IQR) 6.52–7.00 cm, *n* = 14]. In the intervention group, the median distance was 7.15 cm (IQR 6.65–7.40 cm, *n* = 15). One participant in the control group did not undergo a post-procedural CT, *r*_*rb*_ = 0.30, 95% CI = [−0.11, 0.63], *p* = 0.17 (MWU-test).

**Fig. 3:**
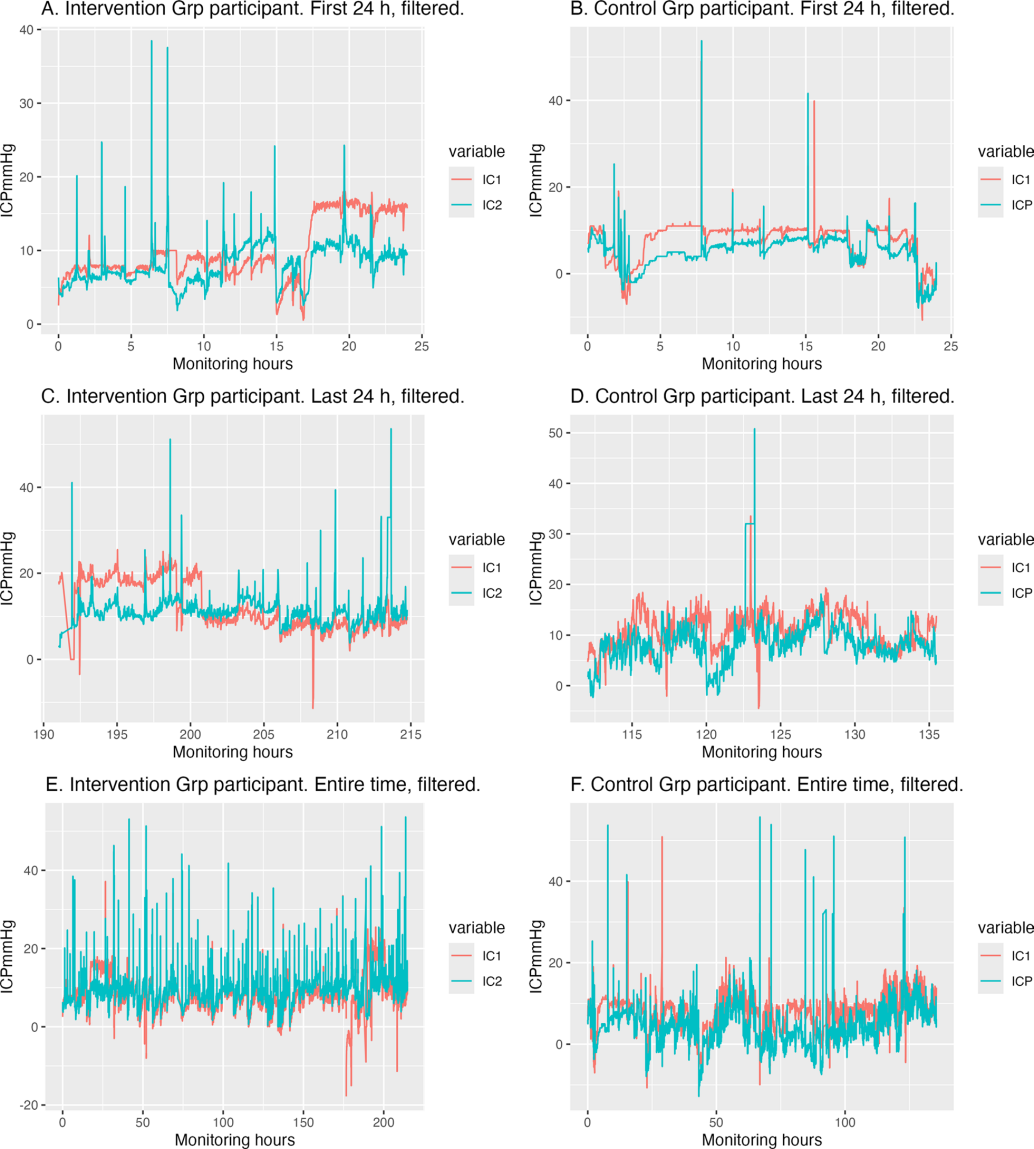
Illustrative time series of ICP measured by an intraparenchymal probe (IC1) and a ventricular drain (IC2), with filtered data. Representative graph for the intervention group and the control group showcased by an individual participant displaying the ICP measurements over the first 24 h (**A**, **B**) and the last 24 h (**C**, **D**) a patient participated in the study. All ICP measurements during the full time of study participation are shown in panel **E** and **F**. The graphs provide an intuitive visual sense of measurements over time showcasing registered IC1 and IC2

To investigate the accuracy of ICP measuring, the median absolute difference between IC1 and IC2 was calculated, where the median in the intervention group (median, non-filtered = 1.07; median, filtered = 1.03, *n* = 15) was lower (*p* < 0.001) than the median in the control group (median, non-filtered = 2.88; median, filtered = 2.84, *n* = 15) (Table 2). This shows that the IC2 measured by the VGuard® system differs less in relation to the reference value, IC1, compared with the IC2 measured by the manual EVD. MWU-test statistics for filtered and non-filtered data: *p* < 0.001, *Z* = −4.54, MWU U = 3.00, *r*_*rb*_ = −0.97, 95% CI = [−0.99, −0.94]. To visualize the ICP measurements (IC1 and IC2) over time, a line-graph was plotted for an individual participant representative for each group (intervention vs. control group) (Fig. 3.)

**Table 2:** Accuracy in measured intracranial pressure (ICP) by calculating the median absolute difference (mm Hg). This shows that the VGuard® system measures ICP with a higher accuracy compared with the manual EVD

Variables	Control, non-filtered^1^	Intervention, non-filtered^2^	Control, filtered^3^	Intervention, filtered^4^
Number of participants	15	15	15	15
Median abs diff (IQR) (mm Hg)^5^	2.88 (2.68–3.61)^6^	1.07 (0.85–1.31)^6^	2.84 (2.51–3.59)^6^	1.03 (0.84–1.28)^6^
Min abs diff (mm Hg)	1.48	0.31	1.47	0.31
Max abs diff (mm Hg)	6.47	1.88	6.35	1.88

^1^Control group showcasing non-filtered data

^2^Intervention group showcasing non-filtered data

^3^Control group showcasing filtered data

^4^Intervention group showcasing filtered data

^5^Median absolute difference between the measured intraparenchymal ICP (IC1) and the measured ventricular ICP (IC2) in mm Hg

^6^*p* < 0.001

**Fig. 4:**
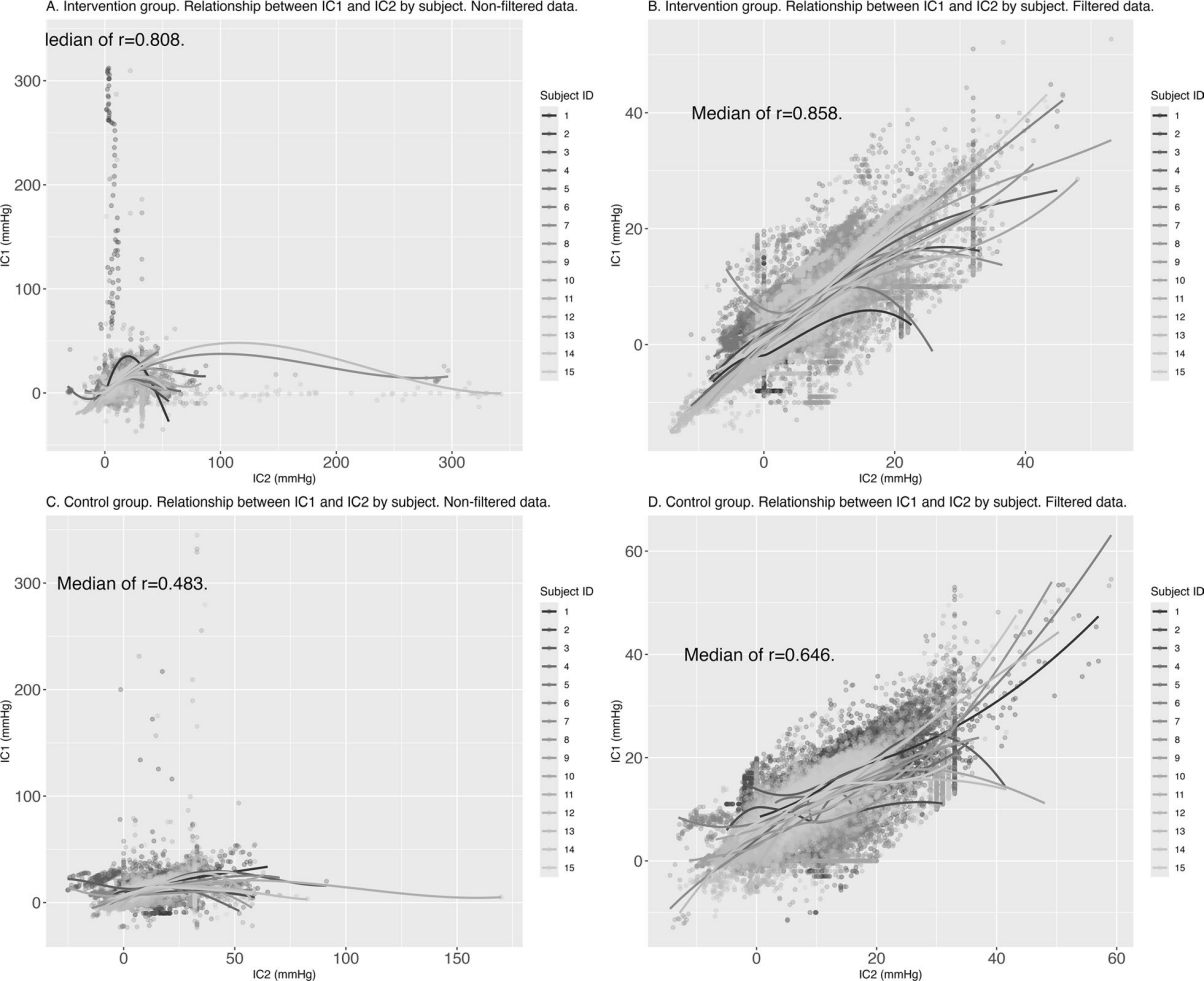
Spearman correlation scatter plots. Spearman correlation scatter plots displaying the correlation (*r*) between the intraparenchymally measured intracranial pressure (IC1) and intraventricularly measured intracranial pressure (IC2) by subject (participant) for both non-filtered and filtered data in the intervention group (**A**, **B**) and the control group (**C**, **D**). The median *r* was calculated using Fisher *Z*-transformation, converting the correlations to *Z*-values and then applying the inverse Fisher *Z*-transformation. All individually calculated *r* values were significant (*p* < 0.05). The median *r* was significantly higher (*p* < 0.05) in the intervention group compared with the control group in both the filtered and non-filtered dataset after applying a MWU-test. Significance level was set to *p* < 0.05

Furthermore, to investigate the correlation between IC1 and IC2, the median Spearman correlation coefficients (median of *r*) were calculated for the respective groups. The median *r* in the intervention group was higher (median of *r*, non-filtered = 0.808; median of *r*, filtered = 0.858) compared with the median *r* in the control group (median of *r*, non-filtered = 0.483; median of *r*, filtered = 0.646). MWU-test statistics for non-filtered data: *p* < 0.001, *Z* = 3.26, MWU *U* = 191, *r*_*rb*_ = 0.70, 95% CI = [0.42, 0.86]. MWU-test statistics for filtered data: *p* < 0.001, *Z* = 3.21, MWU *U* = 190, *r*_*rb*_ = 0.69, 95% CI = [0.40, 0.85]. Individually calculated Spearman correlation coefficients (*r*) were all significant (*p* < 0.05). For visualization of the correlations plotted by the participant, please see Fig. 4.

### Events and Patient Outcomes

No statistically significant differences were found for AEs and SAEs between the intervention group and the control group. The total number of registered AEs in the intervention group were 21 versus 13 in the control group (please see Table 3 for a detailed overview of adverse events). The most common AE observed in both groups was pneumonia (nine in the intervention group and six in the control group). Three device-related issues occurred in the two groups separately, one EVD-occlusion and two EVD-dislodgements in the control group and one EVD-occlusion, one EVD-dislodgment, and one reported technical error E9 in the intervention group. Technical error E9 is reported as power failure. Please see the Supplementary Material for a display of technical errors defined by the manufacturer of the VGuard® system. No SAEs occurred in either group. In either group, no skin infection related to the external sensor was noted. Importantly, since the adverse events are underpowered, the results should strictly be seen as exploratory.

**Table 3:** Adverse events (AE) occurring during study participation. Fisher’s exact test showed no statistically significant differences when comparing the groups

Events	Control group (*n*)	Intervention group (*n*)	Odds ratio [95% CI]; *p*-Value
Cerebral vasospasm	2	3	1.60 [0.15, 22.30]; 1.00
Cardiac complications^1^	1	4	4.84 [0.40, 267.80]; 0.33
Device-related issues^2^	3	3	1 [0.11, 9.06]; 1
Pulmonary edema	0	1	Infinity [0.03, Infinity]; 1
Pneumonia^3^	6	9	2.19 [0.42, 12.38]; 0.47
Thromboembolic event^4^	1	0	0.00 [0.00, 39.00]; 1
Ventriculitis	0	1	Infinity [0.03, Infinity]; 1

^1^Cardiac complications were defined as: atrial fibrillation, left ventricular failure, ventricular tachycardia, receiving pacemaker, heart attack, and premature ventricular contraction

^2^Device-related issues were defined as: occlusion of the external ventricular drain (EVD), dislodgement of the EVD and any type of predefined technical error defined by the manufacturer of the VGuard® system (please see Supplementary Material. In the control group, three device-related issues occurred: one occlusion and two dislodgements. In the intervention group, three device-related issues occurred: one occlusion, one dislodgement, and one technical error E9

^3^Pneumonia was defined as: infectious pneumonia, ventilator-associated pneumonia, and aspiration pneumonia

^4^Thromboembolic events were defined as: venous and pulmonary embolisms as well as sinus thrombosis

Significance level was set to *p* < 0.05

**Fig. 5:**
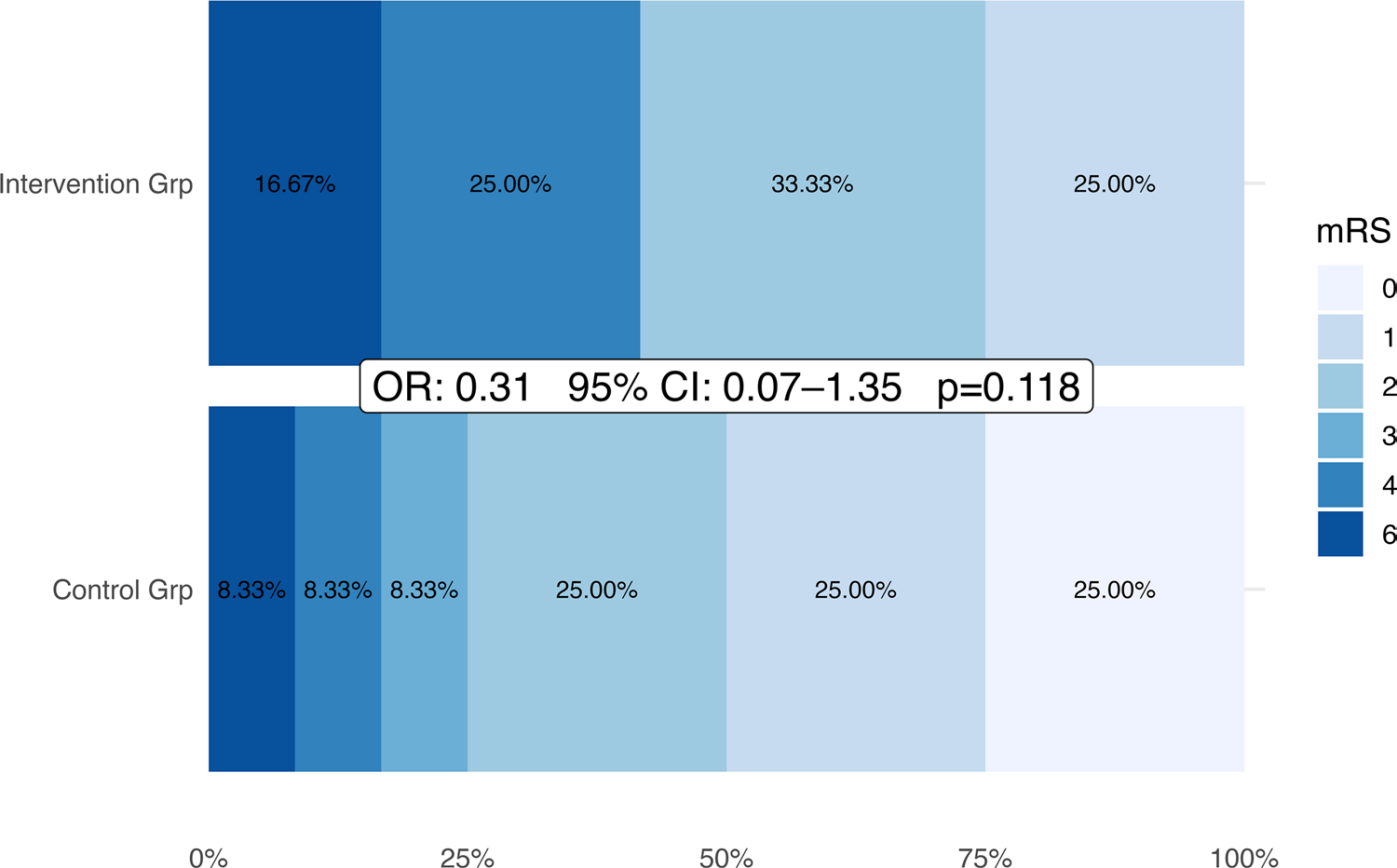
Modified Rankin Scale (mRS). Shift analysis showcasing mRS in both groups with a median of 2 in the respective groups and an IQR of 2–4 in the intervention group (participants receiving the VGuard® system) and 1–2 in the control group (participants receiving the manual external ventricular drainage system). Shift analysis: *n*_*InterventionGrp*_ = 12, *n*_*ControlGrp*_ = 12, improved OR: 0.31, 95% CI = (0.07–1.35), *p* = 0.118 (values > 1 would favor treatment; here, < 1 suggests worse outcomes on treatment, not significant)

There were no statistically significant differences among the variables used to evaluate patient outcomes. The median length of ICU stay was 16 (IQR 11–17) days in the intervention group and 13 (IQR 9–18) days in the control group [*n*_ControlGrp_ = 15, *n*_InterventionGrp_ = 15, *r*_*rb*_ = 0.08, 95% CI = (−0.33, 0.46), *p* = 0.74 (MWU-test)]. The median time with an EVD in both the control (IQR 11−18) and intervention group (IQR 12−17) was 15 days [*n*_ControlGrp_ = 15, *n*_InterventionGrp_ = 15, *r*_*rb*_ = −0, 95% CI = [−0.40, 0.40], *p* = 1 (MWU-test)]. Mortality, GOSE, and mRS were collected at a follow-up ranging between 6 months and 18 months after the patient participated in the study. The median GOSE was 6 (IQR 4–6) in the intervention group versus 7 (IQR 5–7) in the control group [*n*_InterventionGrp_ = 12, *n*_ControlGrp_ = 12, *r*_*rb*_ = −0.33, 95% CI = (−0.67, 0.13), *p* = 0.18 (MWU-test)]. Concerning mRS, the median was 2 (IQR 2–4) in the intervention group and 2 (IQR 1–2) in the control group [shift analysis: *n*_ControlGrp_ = 12, *n*_InterventionGrp_ = 12, improved odds ratio (OR): 0.31, 95% CI = [0.07–1.35], *p* = 0.118 (values > 1 would favor treatment; here, < 1 suggests worse outcomes on treatment, not significant)] (Fig. 5). In the intervention group, two participants versus one participant in the control group died at follow-up (*n*_InterventionGrp_ = 12, *n*_ControlGrp_ = 12, OR = 2.13, 95% CI = [0.10, 141.26], *p* = 1). In total, three participants in both the control and intervention group were lost at follow-up. The outcome results should strictly be seen as exploratory and hypothesis driven rather than confirmatory, since those were underpowered.

## Discussion

The results of this randomized study indicate that the VGuard® system is a promising tool for automated ICP measurement and ventricular drainage, with the main finding being improved accuracy in measured ICP when compared with the manual EVD. Theoretically, this could lower the risk of complications due to manual management of the EVD. For example, under- or overdrainage of CSF, which may lead to intracerebral hemorrhage and coma. Automated health care systems, including the VGuard® system, could reduce work load in the NICU, improve continuous accuracy, and potentially reduce complications associated with manual systems.

To our knowledge, only one other device was created with the purpose of following the patient’s head vertically [37]. This was achieved by mounting the EVD stand to the patient’s bed, enabling the pressure transducer to constantly be kept at level with the foramen of Monro. However, this mounting device only works if the patient’s head is resting in bed, in line with the mattress surface [37]. Since the VGuard® system does not depend on the head being fixed to the bed, the VGuard® system may be a solution more easily adopted to clinical scenarios encountered in NICU management.

No significant differences in AEs and SAEs were seen between the intervention group and the control group. Nevertheless, we reported and presented all types of AE and SAE for transparency and for future research. One AE occurring solely in the intervention group was technical error E9, which indicates power failure. This could possibly lead to faulty measured ICP and thereby faulty CSF drainage, leading to consequences such as hemorrhagic insults and hydrocephalus. However, the reported error message did not lead to any clinical consequences for the included participant, arguing that the VGuard® system is a safe and reliable clinical monitoring tool. Additionally, the VGuard® system has a built in alarm system, notifying the care staff if a technical error occurred, minimizing the risk of an error leading to an event where the patient is harmed in any way. There were no significant differences between the groups in any outcome variable; however, as discussed in previous studies, an automated ventricular system could lead to fewer secondary complications due to immobility [25, 26]. Earlier mobilization may lead to shorter length of ICU stay, as well as less re-hospitalization, as shown in previous studies [38–42]. Given the small, underpowered sample, robust conclusions cannot be drawn. Accordingly, adverse events and outcomes are exploratory, not confirmatory. However, our present results may contribute to future studies of automated systems, such as the VGuard®, in the NICU. Furthermore, automated EVD systems may support improved patient management by reducing accidental episodes in which the drain is not correctly zero-leveled. Such misalignment can theoretically result in under- or overdrainage of CSF, possibly leading to consequences such as intracranial hemorrhage or coma. In current practice, the zero-leveling is performed manually by nursing staff every time the patient’s head position changes relative to the transducer. This procedure is susceptible to human error—particularly under conditions of high workload, thereby increasing the risk of missed or delayed re-leveling. Automating the zero-leveling process could therefore reduce the risk of complication and improve workflow efficiency, which could yield economic benefits. However, statements about complications, workload and costs are hypothesis-driven and require further research with prospective evaluations.

A strength of this study is the large number of registered monitoring hours (intervention group: 3552 h, control group: 2712 h), which enabled thorough assessment of the ICP measuring accuracy. Another strength is that this study is randomized, contrary to previous studies on the topic of automated EVD systems [23–26]. The randomization prevents the study from being influenced by bias and confounding variables. Additionally, both the filtered and non-filtered dataset were included, which provides transparency and credibility. The filtering thresholds were chosen on the basis of previous literature in combination with clinical experience (please see the Methods section for more details regarding the chosen thresholds). One could argue that the ICP threshold is not optimally defined and could be either too low or too high. However, evidence to support alternative limits is sparse, as contemporary ethical standards largely preclude invasive studies in healthy individuals to define normal ICP ranges. Consequently, it is difficult to argue against the thresholds set in this study. Furthermore, recent research has questioned the concept of a fixed “normal” ICP range. A more individualized, multifactorial approach has been proposed, as studies demonstrate that ICP varies with age and intracranial compliance, and individual differences in cerebrospinal fluid and cerebral blood flow dynamics [43–45]. This highlights the inherent difficulty in defining true ICP thresholds. As any selected thresholds may be too high or too low, it is acknowledged as a potential limitation. However, the primary conclusions do not rely exclusively on the filtered dataset, as the unfiltered data also support the findings. Additionally, because all participants were managed in an environment with continuous 24-h nursing supervision, substantial sustained discrepancies in ICP measurements are unlikely to occur. The only circumstance in which marked transient differences may arise is during recalibration of the intraparenchymal sensor. As both groups used the same intraparenchymal sensor, any device-related measurement error inherent to the sensor would be shared equally between groups. Some of the excluded values may be attributable to clinical events, including rapid changes in head position, opening of drains, and potentially drain-related complications such as dislodgement or occlusion. In addition, we are aware that the Moberg monitor introduced electronic noise. Consequently, a subset of the excluded values is likely related to this artifact.

A strength of this study is the treatment protocol. All NICU patients received a Spiegelberg ventricular probe, which avoids incorrectly measured intraparenchymal ICP due to shifted atmospheric pressure, thus providing the reference ICP value (IC1) with a higher accuracy. This stands in comparison with other intraparenchymal ICP sensors that do not compensate for the shifted atmospheric pressure [16, 46–48]. However, while the Spiegelberg sensor is specifically designed to minimize drift-dependent error and reduce inter-sensor variation, residual variation remains, largely reflecting true physiological variability. The drift-dependent error and inter-sensor variations of the Spiegelberg probe have been investigated. In an article by Czosnyka et al. (1997), they report that the long-term zero drift was less than 0.7 mm Hg in the Spiegelberg monitor, with no temperature drift [49]. Lang et al. (2003) showed that the absolute difference between the Spiegelberg monitor and the intraventricular pressure was less than ±3 mm Hg in 99.6% and less than ±2 mm Hg in 91.3% of readings. An Altman–Bland bias plot revealed good agreement between the two methods, with an average bias of 0.5 mm Hg [46]. In an article by Yau et al. (2000), they demonstrate that intracranial pressure measured using the Spiegelberg probe displayed a linear correlation with ICP measured using the standard intraventricular fluid-filled catheter (*r*^2^ = 0.9846, *p* < 0.001; average bias −0.74 mm Hg), as well as with ICP measured using the Codman intraparenchymal strain-gauge sensor (*r*^2^ = 0.9778, *p* < 0.001; average bias 0.01) [50]. Additional studies have investigated inter-sensor variation. However, these have not involved the intraparenchymal Spiegelberg probe. As such, their findings are less directly applicable to the present discussion, although they do provide important contextual insight into inter-sensor variability [51, 52]. The reported median absolute difference is small. Based on the previous reported average bias, it is more likely that the reported difference lies outside the inter-sensor variation. However, it is important to acknowledge inter-sensor variation, as well as the potential influence of drift-dependent error and average bias on measured ICP. Although statistically significant within this small study, the clinical relevance of such modest differences in ICP remains to be established in larger cohorts.

A limitation of the study is the small sample size of study participants, thus restricting statistically significant conclusions. Moreover, owing the study not being blinded, the results may be influenced by performance and detection bias. Furthermore, a limitation of the study is that the CSF drainage was not measured, thus limiting proper conclusions to be drawn regarding over- or underdrainage of CSF. Another limitation of this study is that the EVD was not clamped when measuring ICP. It is known that while the EVD is open, ICP measurements can be faulty [53–55]. In our study, the intention was to clamp the EVD before ICP documentation. However, early in the course of the study, we realized that this was not practically feasible in a real-life clinical setting. In previous publications, the median duration of EVD closure lasted for 25 s, providing additional perspective on the difficulty in converting the desired time of EVD clamping to a real-life NICU clinical setting [55, 56]. Importantly, in our study, the EVD was not closed in either group, creating equal settings for the interventional group and the control group, enabling comparisons to be carried out between the groups. Additionally, the demographics of admission diagnoses were similar in both groups, arguing for similar time periods of CSF drainage (open EVD). Nevertheless, the problem of faulty ICP measurements when draining CSF is still valid. Therefore, the authors do not emphasize the exact ICP value monitored via the ventricular sensor. Instead, as stated in the aim, this study focuses on the difference in measured ICP when comparing the two groups, not in-group comparisons. However, in future studies, further investigation of in-group differences regarding IC1 versus IC2 is warranted for additional evaluation of the novel VGuard® system. Additionally, post hoc analyses were performed and not blinded, which is acknowledged as a potential source of bias. The post hoc analysis was performed after completion of the randomized trial. Analyst blinding to treatment assignment was not feasible because treatment identifiers and timing information were required to derive exposures and outcomes. The authors acknowledge the potential for bias. To diminish it, the analytic steps after data lock were prespecified, and the Spearman correlation test was reported alongside the median absolute difference of ICP. Regarding the post hoc outcome analyses, the findings should be interpreted as exploratory rather than confirmatory because the sample size was too small to provide adequate statistical power, and these inherent limitations are acknowledged. Furthermore, it is recognized that the improved accuracy in ICP measuring is modest. This study was not designed to establish clinical superiority of VGuard®, rather, it aimed to assess technical accuracy and clinical feasibility. Larger, adequately powered randomized studies are needed to quantify the magnitude of any accuracy advantage and to determine its relationship to patient outcomes. In future studies, the evaluation of automated ventricular systems is relevant in view of the potential advantages shown by previous studies as well as the present one [23–26]. Clinical safety, indications, and potential benefits of automated ventricular systems should be evaluated in a larger series of NICU patients.

## Conclusions

This is the first study to evaluate the VGuard® system, showing results of increased accuracy in ICP measurement when compared with the manual EVD (*p* < 0.001). The VGuard® system offers an automated EVD solution, representing a step toward increased automation of neurointensive bedside care, something that still remains limited. Owing to the small sample size of the study, the outcome results were underpowered, producing exploratory results rather than confirmatory results. Additional studies are needed to further investigate the potential benefits of automated EVDs, focusing on the effect of the system on outcome measures.

## Supplementary Information

Below is the link to the electronic supplementary material.

Supplementary file1 (TIFF 64 KB)

Supplementary file2 (TIFF 39 KB)

Supplementary file3 (TIFF 42 KB)

Supplementary file4 (TIFF 56 KB)

Supplementary file5 (PDF 134 KB)

Supplementary file6 (CSV 3 KB)

## Data Availability

The datasets generated and/or analyzed during the current study are not publicly available owing to protection of participant privacy and confidentiality but are available from the corresponding author on reasonable request.

## Abbreviations

NICU: Neurocritical care unit
ICP: Intracranial pressure
CSF: Cerebrospinal fluid
EVD: External ventricular drainage
IC1: The intraparenchymally monitored intracranial pressure
IC2: The intraventricularly monitored intracranial pressure

## Declarations

### Trial Registration

NCT05177692; ClinicalTrials.gov (registered 14 December 2021) https://clinicaltrials.gov/study/NCT05177692.
