# Supplementary figures and images for: Evaluation of a Novel Automated Cerebral Ventricular Drainage System for Intracranial Pressure Monitoring and Cerebrospinal Fluid Drainage in Neurocritical Care Patients: A Prospective, Randomized Clinical Study
Source: Neurocrit Care. 2026 Mar 23;45(1):368–81. doi: 10.1007/s12028-026-02477-4 (PMC13369641; doi:10.1007/s12028-026-02477-4)

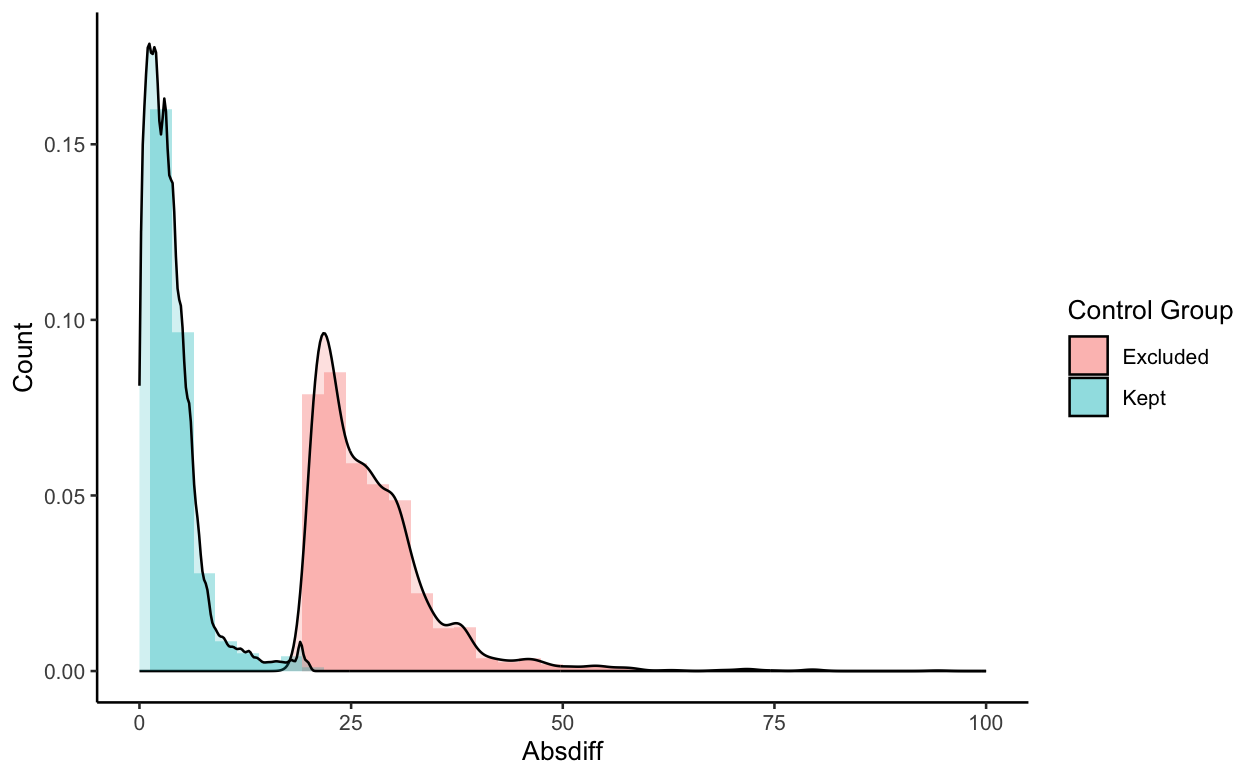

Supplement: Supplementary file 1 — Supplementary file1 (TIFF 64 KB) [file 12028_2026_2477_MOESM1_ESM.tiff]

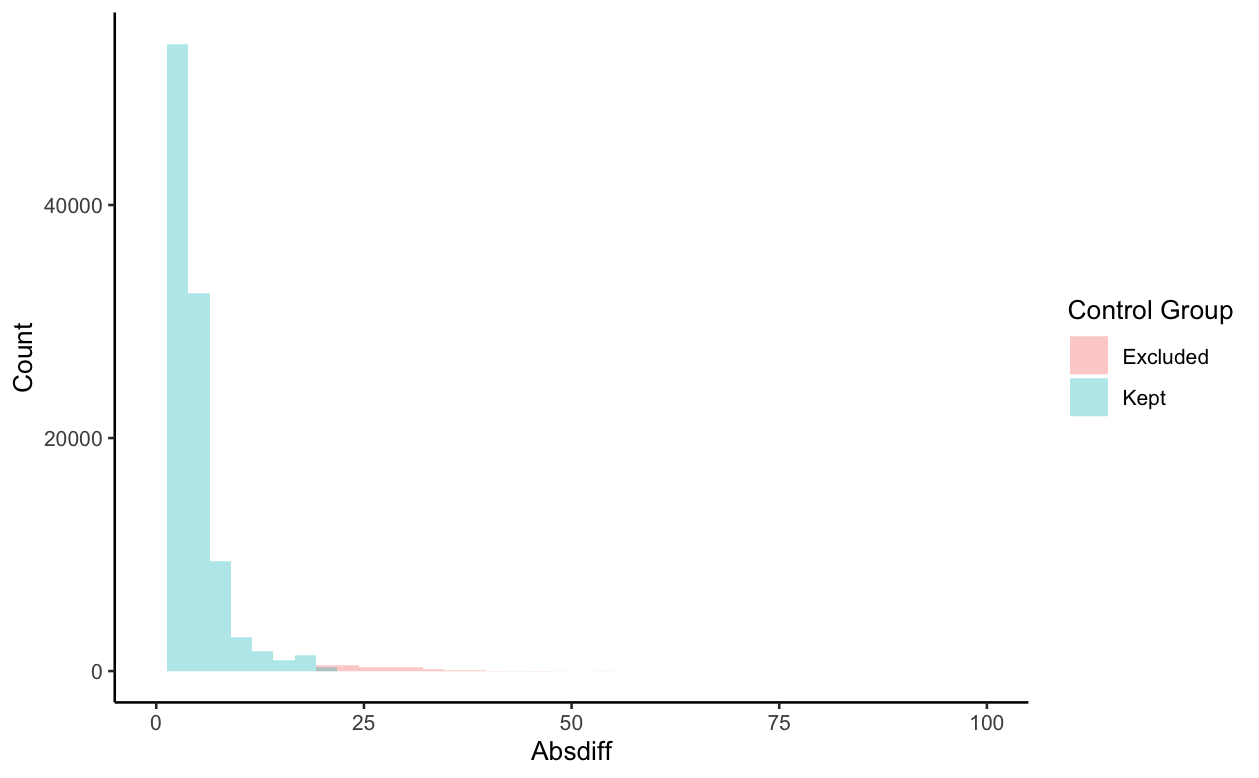

Supplement: Supplementary file 2 — Supplementary file2 (TIFF 39 KB) [file 12028_2026_2477_MOESM2_ESM.tiff]

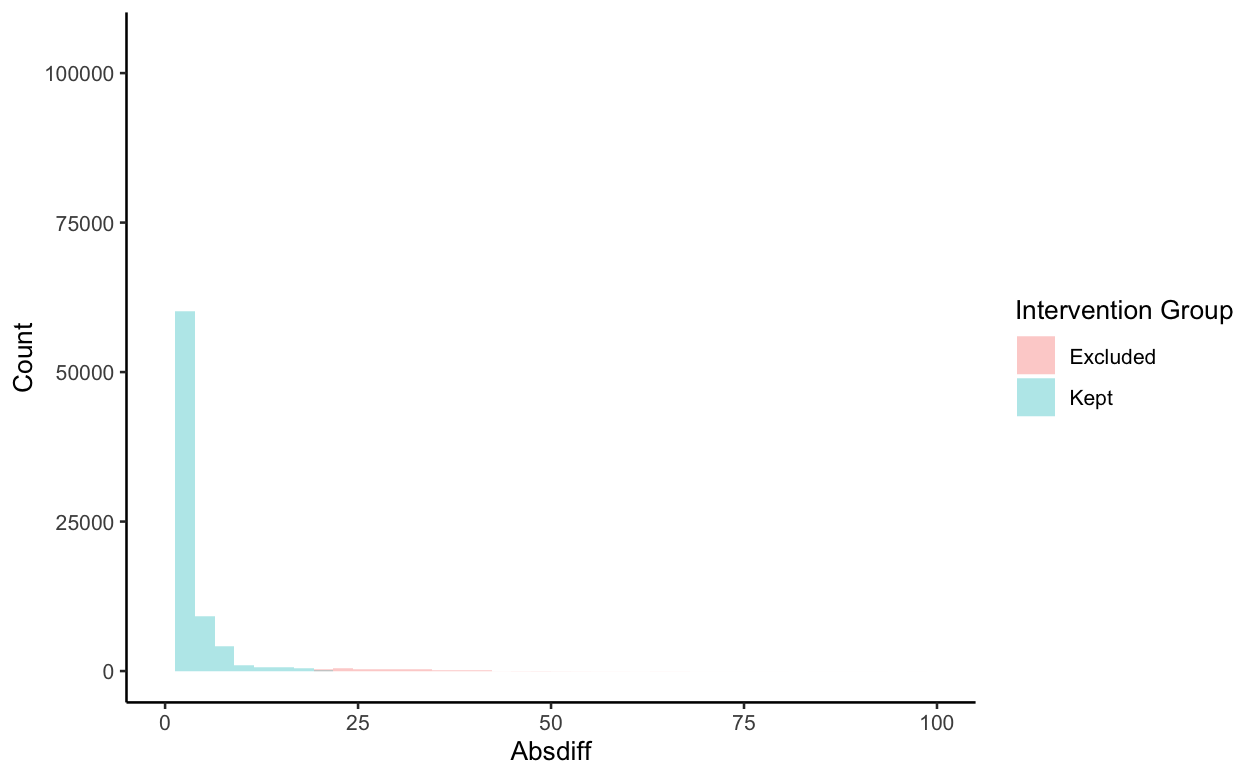

Supplement: Supplementary file 3 — Supplementary file3 (TIFF 42 KB) [file 12028_2026_2477_MOESM3_ESM.tiff]

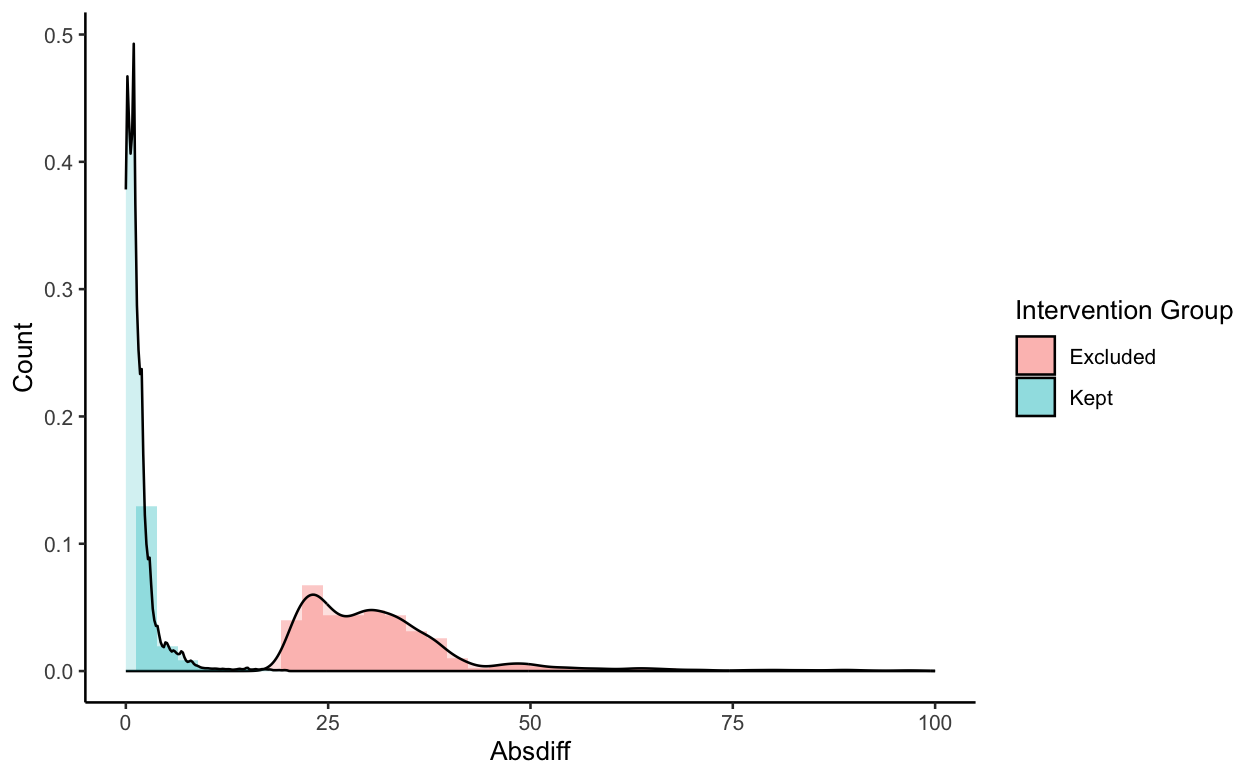

Supplement: Supplementary file 4 — Supplementary file4 (TIFF 56 KB) [file 12028_2026_2477_MOESM4_ESM.tiff]
