# Supplementary material for: Evaluation of a Novel Automated Cerebral Ventricular Drainage System for Intracranial Pressure Monitoring and Cerebrospinal Fluid Drainage in Neurocritical Care Patients: A Prospective, Randomized Clinical Study
Source: Neurocrit Care. 2026 Mar 23;45(1):368–81. doi: 10.1007/s12028-026-02477-4 (PMC13369641; doi:10.1007/s12028-026-02477-4)
Supplement: Supplementary file 5 — Supplementary file5 (PDF 134 KB) [file 12028_2026_2477_MOESM5_ESM.pdf]

1 ALARMS

The alarm function of the system consists of icons, a Mute/Reset button and red and yellow LEDs on the lower part of the front panel. There is also an internal loudspeaker for the audible alarm

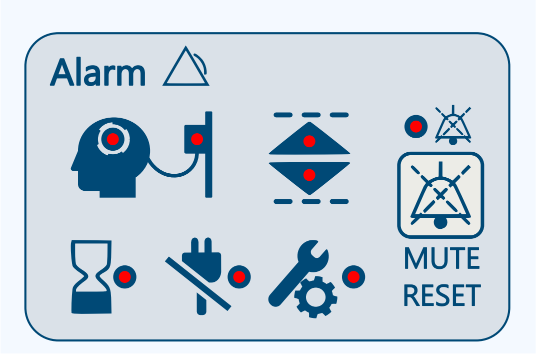

Figure 1 The alarm user interface

If an alarm condition is detected, the corresponding alarm LED will light up immediately and if treatment is active there will also be an audible alarm.

Alarm priority	*LED Colour*	*Style*
High	Red	Flashing
Medium	Yellow	Flashing
Low	Yellow	Constant

Table 1-1 Alarm priority LED behaviour

Mute alarm: Pressing the Mute/Reset button mutes the alarm for 2 minutes.

Clear alarm: If the alarm condition is no longer present the alarm is either automatically cleared or the user can clear it with a long press of the Mute/Reset button.
While clearing the alarm with the Mute/Reset button an internal alarm error number is displayed. This internal alarm number may also be displayed when the alarm is active by simultaneously pressing the + and – button.
When the alarm has been cleared treatment continues if treatment was active when the alarm triggered.

1.1 LIST OF ALARMS

Alarms are cleared in one of the following ways: (see table below)

- P = Power Cycle is required to clear the alarm.
- C = Confirm the alarm by a long press on Mute/**Reset** button to clear the alarm.
- A = Automatic Reset. Alarm will be cleared when the actual cause has disappeared or been removed e.g., obstacle is removed, temperature has dropped.

Table 1-2 Alarm descriptions.

Alarm type	LED ind.	Alarm Condition	Possible cause	Impact on treatment	Reset Criteria	Reset
Obstacle	Flashing:<br>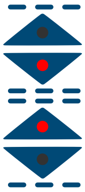	Movement is hindered	An obstacle is blocking movement, or the required movement is outside of actuator's range.	Treatment continues	Remove any obstacles and/or manually adjust the Main Unit mounting position on the stand.	A
Cannot Reach Zero Level Within Time Limit	Flashing:<br>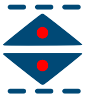	It takes too long to reach zero level (>1 minute).	Patient moves too fast,<br>Technical failure in Main Unit,<br>Obstacle alarm not resolved within 1 minute.	Treatment continues	Zero-level reached.<br>Remove obstacle and/or manually adjust the Main Unit mounting position on the stand.	A
Sensor coming loose	Flashing:<br>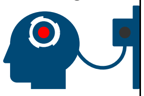	Proximity sensor reports distance in front of sensor.	The patient end of the sensor has most likely fallen out of the sensor holder.	Stops treatment temporarily	Attach the sensor to the sensor holder.<br>Clear alarm by mute/reset button to resume treatment.<br>Replace sensor if alarm continues to trigger on treatment restart.	C
Sensor error general	Flashing:<br>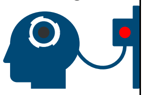	No data, corrupt data or data out of range.	Elevator end of sensor not connected or faulty sensor	Stops treatment temporarily	Make sure the sensor is correctly connected to the machine.<br>Clear the alarm to resume treatment.<br><br>Replace sensor if alarm still can't be cleared.	C or A
Technical error	Flashing:<br>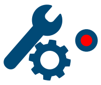	Internal failure	Internal component failures, high temperatures, moisture in main unit, hardware malfunction,<br>missing/faulty memory	Stops treatment	Reset the alarm by mute/reset button or power cycle. Contact technician if error reoccurs or alarm cannot reset.	C or P
Technical error - logging	Solid:<br>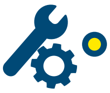	Internal failure	Error in logging, for example SD card missing or real time clock reset.	Treatment continues	Power cycle. If error persists, contact technician.	P
Loss of power	Flashing:<br>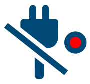	Power failure	Power cord is disconnected<br>Power outage<br><br>NOTE: Due to technical limitations (limited power) the sound of this alarm is different. When power is restored, the sound is restored.	Stops treatment	Connect to power if disconnected.<br><br>If there is a power loss, the back-up alarm will sound for 2 minutes. If the system has power restored within 10 minutes of the power loss, the Loss of Power alarm will be triggered as the system is powered.	C
Warning – Sensor max usage time	Solid or flashing:<br>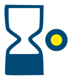	Sensor will soon reach the max usage time	Solid:<br>Sensor has been used for more than 29 days.<br><br>Flashing:<br>Sensor has been used for more than the max time, 30 days.	Treatment continues	Solid:<br>No action needed. The alarm can only be reset by replacing the sensor with a new one.<br><br>Flashing:<br>Stop the treatment and change to a new sensor. After the treatment has been stopped it cannot be restarted until the sensor is replaced with a new one	A
